# Supplementary figures and images for: Identifying bedrest using waist-worn triaxial accelerometers in preschool children
Source: PLoS One. 2021 Jan 28;16(1):e0246055. doi: 10.1371/journal.pone.0246055 (PMC7842939; doi:10.1371/journal.pone.0246055)

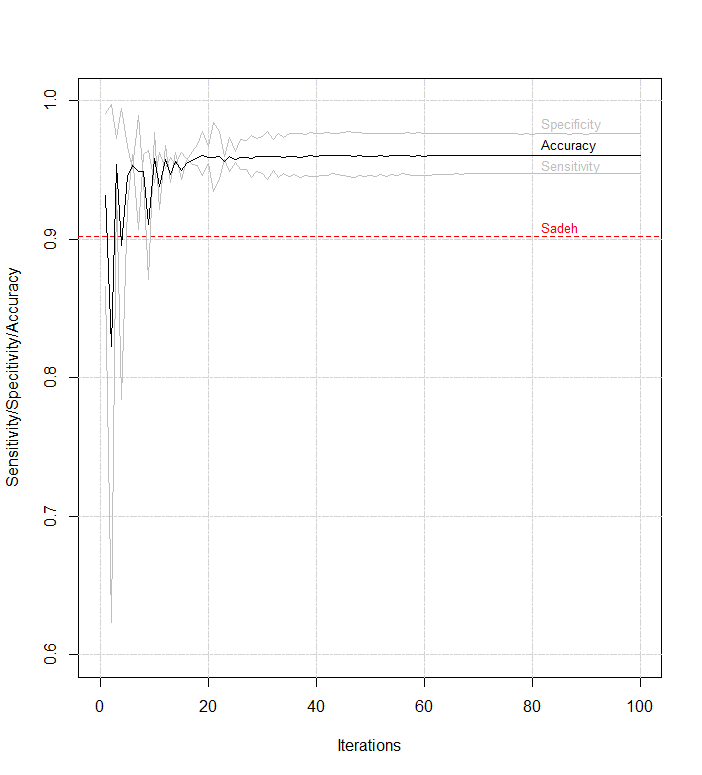

Supplement: S1 Fig — (TIF) [file pone.0246055.s001.tif]
