# Supplementary material for: Identifying bedrest using waist-worn triaxial accelerometers in preschool children
Source: PLoS One. 2021 Jan 28;16(1):e0246055. doi: 10.1371/journal.pone.0246055 (PMC7842939; doi:10.1371/journal.pone.0246055)
Supplement: S1 Table — (DOCX) [file pone.0246055.s002.docx]

**S1 Table**

**Parameters sets used to initialize decision tree (DT) algorithm optimization using Nelder-Mead Simplex procedure.**

|  | **Threshold**  **(counts/min)** | **Bedrest End Trigger**  **(counts/min)** | **Bedrest Start Trigger**  **(counts/min)** | **Block Length**  **(min)** |
| --- | --- | --- | --- | --- |
| *Initial Set 1* | 100 | 800 | 100 | 20 |
| *Initial Set 2* | 20 | 400 | 40 | 30 |
| *Initial Set 3* | 300 | 1500 | 300 | 60 |
| *Initial Set 4* | 60 | 1000 | 200 | 80 |
