# Supplementary material for: Identifying bedrest using waist-worn triaxial accelerometers in preschool children
Source: PLoS One. 2021 Jan 28;16(1):e0246055. doi: 10.1371/journal.pone.0246055 (PMC7842939; doi:10.1371/journal.pone.0246055)
Supplement: S2 Table — (DOCX) [file pone.0246055.s003.docx]

**S2 Table**

**Progression of the optimization of decision tree (DT) algorithm parameters.**

| **Iteration** | **Sensitivity** | **Specificity** | **Accuracy** | **Iteration** | **Sensitivity** | **Specificity** | **Accuracy** |
| --- | --- | --- | --- | --- | --- | --- | --- |
| 1 | 0.8661 | 0.9912 | 0.9314 | 43 | 0.9473 | 0.9763 | 0.9606 |
| 2 | 0.6236 | 0.9971 | 0.8228 | 44 | 0.9468 | 0.9765 | 0.9607 |
| 3 | 0.9364 | 0.9732 | 0.9539 | 45 | 0.9460 | 0.9772 | 0.9605 |
| 4 | 0.7841 | 0.9947 | 0.8956 | 46 | 0.9456 | 0.9778 | 0.9608 |
| 5 | 0.9244 | 0.9673 | 0.9455 | 47 | 0.9447 | 0.9775 | 0.9602 |
| 6 | 0.9614 | 0.9512 | 0.9533 | 48 | 0.9461 | 0.9770 | 0.9603 |
| 7 | 0.9069 | 0.9896 | 0.9494 | 49 | 0.9453 | 0.9766 | 0.9598 |
| 8 | 0.9610 | 0.9434 | 0.9491 | 50 | 0.9461 | 0.9764 | 0.9601 |
| 9 | 0.9644 | 0.8708 | 0.9109 | 51 | 0.9452 | 0.9768 | 0.9598 |
| 10 | 0.9409 | 0.9772 | 0.9581 | 52 | 0.9465 | 0.9763 | 0.9602 |
| 11 | 0.9625 | 0.9219 | 0.9381 | 53 | 0.9456 | 0.9767 | 0.9601 |
| 12 | 0.9510 | 0.9678 | 0.9578 | 54 | 0.9468 | 0.9764 | 0.9603 |
| 13 | 0.9590 | 0.9412 | 0.9469 | 55 | 0.9465 | 0.9763 | 0.9603 |
| 14 | 0.9540 | 0.9624 | 0.9563 | 56 | 0.9475 | 0.9761 | 0.9606 |
| 15 | 0.9628 | 0.9432 | 0.9498 | 57 | 0.9465 | 0.9769 | 0.9606 |
| 16 | 0.9582 | 0.9567 | 0.9550 | 58 | 0.9459 | 0.9764 | 0.9599 |
| 17 | 0.9542 | 0.9636 | 0.9567 | 59 | 0.9464 | 0.9768 | 0.9605 |
| 18 | 0.9531 | 0.9683 | 0.9589 | 60 | 0.9464 | 0.9763 | 0.9602 |
| 19 | 0.9459 | 0.9776 | 0.9606 | 61 | 0.9463 | 0.9766 | 0.9603 |
| 20 | 0.9547 | 0.9667 | 0.9588 | 62 | 0.9466 | 0.9763 | 0.9602 |
| 21 | 0.9347 | 0.9847 | 0.9594 | 63 | 0.9469 | 0.9761 | 0.9603 |
| 22 | 0.9439 | 0.9782 | 0.9601 | 64 | 0.9471 | 0.9762 | 0.9604 |
| 23 | 0.9576 | 0.9598 | 0.9564 | 65 | 0.9470 | 0.9763 | 0.9604 |
| 24 | 0.9487 | 0.9734 | 0.9596 | 66 | 0.9477 | 0.9761 | 0.9606 |
| 25 | 0.9554 | 0.9638 | 0.9574 | 67 | 0.9471 | 0.9762 | 0.9604 |
| 26 | 0.9502 | 0.9718 | 0.9594 | 68 | 0.9474 | 0.9762 | 0.9606 |
| 27 | 0.9505 | 0.9712 | 0.9593 | 69 | 0.9477 | 0.9761 | 0.9606 |
| 28 | 0.9446 | 0.9749 | 0.9585 | 70 | 0.9474 | 0.9763 | 0.9606 |
| 29 | 0.9492 | 0.9728 | 0.9595 | 71 | 0.9475 | 0.9763 | 0.9606 |
| 30 | 0.9479 | 0.9742 | 0.9597 | 72 | 0.9477 | 0.9761 | 0.9606 |
| 31 | 0.9433 | 0.9782 | 0.9597 | 73 | 0.9474 | 0.9763 | 0.9606 |
| 32 | 0.9501 | 0.9722 | 0.9598 | 74 | 0.9477 | 0.9761 | 0.9606 |
| 33 | 0.9450 | 0.9765 | 0.9596 | 75 | 0.9475 | 0.9763 | 0.9606 |
| 34 | 0.9475 | 0.9734 | 0.9589 | 76 | 0.9475 | 0.9763 | 0.9606 |
| 35 | 0.9452 | 0.9765 | 0.9597 | 77 | 0.9475 | 0.9763 | 0.9606 |
| 36 | 0.9467 | 0.9762 | 0.9602 | 78 | 0.9476 | 0.9760 | 0.9605 |
| 37 | 0.9448 | 0.9762 | 0.9594 | 79 | 0.9475 | 0.9763 | 0.9606 |
| 38 | 0.9461 | 0.9756 | 0.9596 | 80 | 0.9475 | 0.9761 | 0.9605 |
| 39 | 0.9452 | 0.9774 | 0.9602 | … | … | … | … |
| 40 | 0.9454 | 0.9762 | 0.9597 | 98 | 0.9475 | 0.9763 | 0.9606 |
| 41 | 0.9462 | 0.9767 | 0.9602 | 99 | 0.9475 | 0.9763 | 0.9606 |
| 42 | 0.9460 | 0.9771 | 0.9603 | 100 | 0.9475 | 0.9763 | 0.9606 |
