# Supplementary material for: Identifying bedrest using waist-worn triaxial accelerometers in preschool children
Source: PLoS One. 2021 Jan 28;16(1):e0246055. doi: 10.1371/journal.pone.0246055 (PMC7842939; doi:10.1371/journal.pone.0246055)
Supplement: S3 Table — (DOCX) [file pone.0246055.s004.docx]

**S3 Table**

**Comparison of DT (decision tree) algorithm performance between vector magnitude (VM) and single-axis (vertical) recordings in the validation group (n = 200).**

|  | **VM** | **Vertical Axis**^a^ | **P-value**^b^ |
| --- | --- | --- | --- |
| Sensitivity | 0.941 ± 0.056  (0.618, 0.999) | 0.936 ± 0.057  (0.605, 0.995) | **< 0.001** |
| Specificity | 0.974 ± 0.038  (0.566, 0.999) | 0.970 ± 0.040  (0.599, 0.997) | **< 0.001** |
| Accuracy | 0.956 ± 0.043  (0.654, 0.994) | 0.952 ± 0.043  (0.680, 0.993) | **< 0.001** |

values are mean ± standard deviation and values in parentheses are ranges.

^a^ data acquired using parameters optimized for VM

^b^ paired t-test between VM and vertical axis
