# Supplementary material for: Identifying bedrest using waist-worn triaxial accelerometers in preschool children
Source: PLoS One. 2021 Jan 28;16(1):e0246055. doi: 10.1371/journal.pone.0246055 (PMC7842939; doi:10.1371/journal.pone.0246055)
Supplement: S4 Table — (DOCX) [file pone.0246055.s005.docx]

**S4 Table**

**Comparison between performance between decision tree (DT) and Sadeh’s algorithm using recordings from vertical axis in the validation group (n = 200).**

|  |  | **DT Algorithm**  **(Vertical Axis)** | **Sadeh’s Algorithm** | **P-value** ^a^ |
| --- | --- | --- | --- | --- |
| Sensitivity |  | 0.936 ± 0.057  (0.605, 0.995) | 0.920 ± 0.041  (0.706, 0.979) | **< 0.001** |
| Specificity |  | 0.970 ± 0.040  (0.599, 0.997) | 0.888 ± 0.064  (0.506, 0.984) | **< 0.001** |
| Accuracy |  | 0.952 ± 0.043  (0.680, 0.993) | 0.902 ± 0.042  (0.599, 0.965) | **< 0.001** |

values are mean ± standard deviation and values in parentheses are ranges

^a^ paired t-test between DT and Sadeh’s algorithm
