# Supplementary material for: Identifying bedrest using waist-worn triaxial accelerometers in preschool children
Source: PLoS One. 2021 Jan 28;16(1):e0246055. doi: 10.1371/journal.pone.0246055 (PMC7842939; doi:10.1371/journal.pone.0246055)
Supplement: Attachment — Submitted filename: Response to reviewers comments-MSB-final.docx [file pone.0246055.s006.docx]

**Response to Editor and Reviewers**

We thank the Editor and the Reviewer for their time and effort spent reviewing our manuscript and providing us with excellent suggestions and comments. We hope that our responses address the Reviewer’s comments satisfactorily.

*Please note that page numbers reflect those on the redlined document rather than the clean document file.

**Reviewer #1:**  Authors have shared an interesting article about the validation of an adapted algorithm for identification and differentiation of bedrest and wake periods using wait-worm triaxial accelerometers. The work is well structured and includes comprehensive description of methodology and statistical analyses.

Response: Thank you very much.

1. My biggest concern regards to the fairness of the comparison approach with other methods, in particular to what respects to the omission of the role that the database variability effect might play on the results, possibly penalizing performance of the other methods. In effect, authors openly admit, and experimental results also confirm, that usage of the proposed DT(-Youth) algorithm on different age groups (in this case, preschool vs older children) does require reconfiguration, namely different values for the four parameters (block length, threshold, bedrest-start trigger, and bedrest-end trigger) guiding the decision algorithm. Given for example, the benchmarked Sadeh's algorithm was probably developed based on data other than the Growing Right Onto Wellness dataset used in this study (meaning different subjects and signal characteristics), it is somehow expected that performance could drop when comparing it with an algorithm that was optimized for the mentioned dataset. A more fair comparison would be to use a third, completely independent dataset to all the involved algorithms in the comparison, to test the real generalization capabilities of each one.
   Authors should develop into this point, and at the very least include the issue into the discussion and address it as a possible limitation.

Response. We agree with the Reviewer. Having an independent dataset would be an excellent addition to the study, but we did not have such a dataset available. As the Reviewer suggested, we added this concern to the study limitations. We also included the Reviewer's suggestion as one of the directions for future studies.

Lines 422-425. *Better performance of DT than Sadeh's algorithm could be expected since Sadeh's algorithm was developed in a different population and using a different study protocol [26].* *A more objective comparison of algorithms' generalization capacity would be achieved using an independent* *preschool-age child dataset.*

Lines 476-479. *The DT should also be validated in other studies with an independent sample of preschool-age children performed in a different environment. However, the DT allows users to adapt it for bedrest identification in various populations and/or using different accelerometers.*

Lines 464-467. *Future studies should be performed in diverse populations to test and calibrate the DT against PSG and compare it with other automatic algorithms that use different accelerometry data measures such as arm angle or gravitational acceleration data expressed in grams [16].*

2. Authors should also elaborate more on the specific differences between the current version of the DT algorithm proposed and the previously reported, here referred as DT-Youth. It is just about finding a different set of configuration parameters optimized for each targeted age group for the same algorithm? Or are the two methods intrinsically different?

Response. As the Reviewer pointed out, the DT algorithm is based on the DT-Youth. The significant enhancements are using vector magnitude (VM) and using data from several days of recordings.

Lines 411-419.  *The current study took advantage of technological advancements and used VM calculated from counts recorded by three distinct axes to measure movement. We compared the DT performance to identify bedrest for VM and single-axis (vertical) recordings in the validation group and found that both sensitivity and specificity were higher for VM than single-axis data. We recommend that studies assessing bedrest/sleep in preschool children using triaxial accelerometers utilize VM rather than single-axis data. Another advantage of using the modified algorithm is its ability to identify short naps (30-60 min) usually occurring during the day and using data from several days of accelerometer recordings.*

3. Necessity to reparametrize the algorithm on each case could be seen as an additional limiting factor. Apparently every potential user would have to run an optimization procedure similar to the one described by the authors to discover the optimal parameters for their specific local database, which seems rather unpractical, unless any general rule-of-thumb can be derived a priori to find an appropriate set.

Response. The study's goal was to modify the existing algorithm that could be used in healthy 3-6-year-old children. We believe that the algorithm we propose offers robust performance in healthy preschool children. Using the set of parameters (*block length*, *threshold*, *bedrest-start trigger,* and *bedrest-end trigger*) has been validated in youth (ref #25) and adults (ref #30). The optimal values for these parameters are default in the algorithm's package. However, the program allows investigators to change the parameters' value if they choose.

Lines 473-479: *Second, the study results are specific to 3-6 years old, healthy children. However, we believe the set of parameters (block length, threshold, bedrest-start trigger, and bedrest-end trigger) optimized in this study ensures very good DT performance in all preschool children. The DT should also be validated in other studies with an independent sample of preschool-age children and performed in a different environment. However, the DT allows users to adapt it for bedrest identification in various populations and/or using different accelerometers.*

4. An additional point that requires further clarification is that one of the main reasons motivating this work is that, to the authors' knowledge, no accelerometry-based algorithm validation studies in healthy preschool children have been reported" (lines 77-78). What are the reasons for which authors have opted not to include the work referenced in [9] into this discussion?

Response. Thank you for reading our manuscript in detail. The reason for not including Belanger et al. (reference 9) study was that it was performed using an Actiwatch accelerometer that uses recordings from a single axis. The results (counts) are not comparable with results from other accelerometers, including tri-axial accelerometers. In other words, Belanger’s algorithm(s) cannot be used directly for processing data from the Actigraph-GT3X accelerometer used in our study. We used Actigraph because it generates data in g-units, and it is triaxial, allowing the calculation of vector magnitude (VM) useful in assuming physical activity. Actigraph is used in many US studies in children and adults, including the National Health and Nutrition and Examination Survey (NHANES). Relatively widespread use of this accelerometer makes it easier to compare results among studies.

We clarified the statement and included additional information about reference 9 (Belenger et al. 2013).

Lines 79-85: *Recently, Belanger et al. [9] developed two algorithms for the wrist-worn and ankle-worn single-axis Actiwatch accelerometer using data from 2-5 years old children (n=12) during nighttime sleep. However, no triaxial accelerometer-based algorithm validation studies in healthy preschool children have been reported to our knowledge. The present study aims to fill a gap in the literature by identifying bedrest periods that include nighttime sleep and daytime naps from data from accelerometers collected for several days* *with a high degree of accuracy.*

Minor comments:

1. Though Table 4 suggest that no abrupt differences exist in the balance of cases among the two categories considered (bedrest, wake), it would be nice to mention the exact amount of cases on the development and validation subsets, according to the gold standard, in this case, the expert's classifications. If classes would result unbalanced then adding kappa to the reported list of performance statistics might be a better choice than accuracy, as kappa compensates for agreement due to chance, i.e. in case the a priori probability of one class if significantly bigger than the other. It would also allow comparison of the kappa agreement reported to evaluate the expert's inter-scorer reliability.

Response. As suggested, we added information about bedrest and wake duration from the visual identification to Table 4 (the relevant part of the table is included below). The differences between the visual classification, DT, Sadeh’s, and Parental survey did not suggest any unexpected differences between the methods. Therefore, we did not perform further statistical analyses. However, if the Reviewer would recommend performing additional statistical analyses, we could include such analyses either in the manuscript or supplementary materials. Also, the agreement between the raters was assessed using accuracy and kappa coefficient, and the agreement was very good.

Lines 325-326: *The inter-rater agreement was very good (mean sensitivity, specificity, and accuracy were >0.98 and κ (kappa) = 0.99).*

Table 4. Characteristics of bedrest and wake periods identified using DT, Sadeh’s algorithm, and parental survey in the validation group (n=200).

|  | **DT** | **Sadeh’s algorithm** | **Parental survey** | **Visual identification** | **P-value** |
| --- | --- | --- | --- | --- | --- |
| Bedrest  [min/day] | 564 ± 77  (379, 719) | 604 ± 80  (379, 813) | 650 ± 81  (450, 960) | 575 ± 80  (287, 734) | <0.001^a,b,c,d,e,f^ |
| Wake  [min/day] | 680 ± 112  (24, 876) | 640 ± 112  (181, 154) | - | 668 ± 124  (199, 992) | <0.001^a,c,e^ |

2. There is possibly a typo when referencing to Fig. 2 in line 334, which I think it should be Fig. 3 instead.

Response. Thank you. We did not correct this error because Figure 3 was omitted from the revised manuscript, as suggested by the Reviewer in minor comment 3 (below).

3. In addition, I would suggest reconsidering Fig.3 in general and stick reporting to the Area Under to ROC Curve for the final optimum point for the different algorithms, as there is no much interest in reporting the intermediate values during the optimization process. Usually one reports ROC curves to compare different operating final points of an algorithm. Regardless, if decided to report (or display) the results in both the development and validation subsets, identify them more clearly. Now assuming the big plot corresponds to development and the smaller one to the validation set.

Response**.** We agree. We have omitted Figure 3 in the revised manuscript.

**Editorial comments.**

1. Please ensure that your manuscript meets PLOS ONE's style requirements, including those for file naming.

Response. The manuscript meets PLOS ONE's style requirements.

2. Please ensure that you refer to Figure 3 in your text as, if accepted, production will need this reference to link the reader to the figure.

Response. Figure 3 was omitted in the revised manuscript, as recommended by the Reviewer.

3. We note that you have stated that you will provide repository information for your data at acceptance. Should your manuscript be accepted for publication, we will hold it until you provide the relevant accession numbers or DOIs necessary to access your data. If you wish to make changes to your Data Availability statement, please describe these changes in your cover letter and we will update your Data Availability statement to reflect the information you provide.

Response. The relevant data files (with DOIs) are now available in a public repository:

<https://figshare.com/articles/dataset/Train_Files/12904124>

<https://figshare.com/articles/dataset/Test_Files/12904127>

4. Thank you for stating in your Funding Statement:

"This research was supported by the National Institutes of Health (NIH) grants HL103620 and HL103561 from the National Heart, Lung, and Blood Institute and the Eunice Kennedy Shriver National Institute of Child Health and Development and the Office of Behavioral and Social Sciences Research: [https://www.nih.gov/](https://nam12.safelinks.protection.outlook.com/?url=https%3A%2F%2Fwww.nih.gov%2F&data=04%7C01%7Cmaciej.buchowski%40vumc.org%7C8916981c762c4607df6a08d8988390e9%7Cef57503014244ed8b83c12c533d879ab%7C0%7C1%7C637427038130411520%7CUnknown%7CTWFpbGZsb3d8eyJWIjoiMC4wLjAwMDAiLCJQIjoiV2luMzIiLCJBTiI6Ik1haWwiLCJXVCI6Mn0%3D%7C1000&sdata=YcaT3Jrp%2BGh2pio4O3IOhj%2BWhhwU6msWrVYVwyGAdJY%3D&reserved=0). Additional funds were provided by grants DK058404 and DK20593 from the National Institute of Diabetes and Digestive and Kidney Diseases: [https://www.nih.gov](https://nam12.safelinks.protection.outlook.com/?url=https%3A%2F%2Fwww.nih.gov%2F&data=04%7C01%7Cmaciej.buchowski%40vumc.org%7C8916981c762c4607df6a08d8988390e9%7Cef57503014244ed8b83c12c533d879ab%7C0%7C1%7C637427038130421514%7CUnknown%7CTWFpbGZsb3d8eyJWIjoiMC4wLjAwMDAiLCJQIjoiV2luMzIiLCJBTiI6Ik1haWwiLCJXVCI6Mn0%3D%7C1000&sdata=i7OASrMdTv0XgBRg4Vr9KrM1gQtBw%2F3OdGH%2BIi7wR6Q%3D&reserved=0). The REDCap Database was supported by TR000445 from the National Center for Advancing Translational Sciences: [https://www.nih.gov/](https://nam12.safelinks.protection.outlook.com/?url=https%3A%2F%2Fwww.nih.gov%2F&data=04%7C01%7Cmaciej.buchowski%40vumc.org%7C8916981c762c4607df6a08d8988390e9%7Cef57503014244ed8b83c12c533d879ab%7C0%7C1%7C637427038130421514%7CUnknown%7CTWFpbGZsb3d8eyJWIjoiMC4wLjAwMDAiLCJQIjoiV2luMzIiLCJBTiI6Ik1haWwiLCJXVCI6Mn0%3D%7C1000&sdata=i7OASrMdTv0XgBRg4Vr9KrM1gQtBw%2F3OdGH%2BIi7wR6Q%3D&reserved=0). WJH was supported by K23 HL127104 grant from the National Heart Lung and Blood Institute: [https://www.nih.gov/](https://nam12.safelinks.protection.outlook.com/?url=https%3A%2F%2Fwww.nih.gov%2F&data=04%7C01%7Cmaciej.buchowski%40vumc.org%7C8916981c762c4607df6a08d8988390e9%7Cef57503014244ed8b83c12c533d879ab%7C0%7C1%7C637427038130431508%7CUnknown%7CTWFpbGZsb3d8eyJWIjoiMC4wLjAwMDAiLCJQIjoiV2luMzIiLCJBTiI6Ik1haWwiLCJXVCI6Mn0%3D%7C1000&sdata=n6PbqLK5IPDyCyukoOL3xzjYde5yPEQVEwKgQeZUdHI%3D&reserved=0).
The funders had no role in study design, data collection and analysis, decision to publish, or preparation of the manuscript."

i) Please provide an amended statement that declares *all* the funding or sources of support (whether external or internal to your organization) received during this study, as detailed online in our guide for authors at [http://journals.plos.org/plosone/s/submit-now](about:blank).  Please also include the statement “There was no additional external funding received for this study.” in your updated Funding Statement.

ii) Please include your amended Funding Statement within your cover letter. We will change the online submission form on your behalf.

Response. Updated funding statement is included in the cover letter.
