# Supplementary material for: Identifying bedrest using waist-worn triaxial accelerometers in preschool children
Source: PLoS One. 2021 Jan 28;16(1):e0246055. doi: 10.1371/journal.pone.0246055 (PMC7842939; doi:10.1371/journal.pone.0246055)
Supplement: Attachment — Submitted filename: Response to reviewers comments-01-09-2020-MSB.docx [file pone.0246055.s007.docx]

**Response to Editor and Reviewers**

We thank the Editor and the Reviewer again for their time and effort spent reviewing our manuscript and providing us with additional suggestions and comments. We hope that our responses address the Reviewer's comments satisfactorily.

*Please note that page numbers reflect those on the redlined document rather than the clean document file.

**Reviewer #1:** Regarding Table 4, and excluding the new additions regarding Visual identification, I have noticed that numbers regarding remaining categories have changed with respect to the previous version of the manuscript, and also with the parts of Table 4 included in the response to the reviewers.

Please review it carefully, and if applies, explain the reasons behind these changes and modify the corresponding parts in the manuscript text accordingly.

Response: Thank you very much for pointing out this discrepancy. We apologize for the lack of transparency.

The visual identification of bedrest was performed using a labeling software (available at  [https://github.com/shi-xin/actigraph_labeler](https://nam04.safelinks.protection.outlook.com/?url=https%3A%2F%2Fgithub.com%2Fshi-xin%2Factigraph_labeler&data=04%7C01%7Cmaciej.buchowski%40Vanderbilt.Edu%7C7c07e8a1117f41b8191208d8b3623cbc%7Cba5a7f39e3be4ab3b45067fa80faecad%7C0%7C0%7C637456581800755099%7CUnknown%7CTWFpbGZsb3d8eyJWIjoiMC4wLjAwMDAiLCJQIjoiV2luMzIiLCJBTiI6Ik1haWwiLCJXVCI6Mn0%3D%7C1000&sdata=HF5744QUxBlWMagJTGQYWJSGhYUcOOMKj3YeXNLRHaY%3D&reserved=0)), which enabled the user to discard data at the beginning and end of each Actigraph file. The previous Table 4 was produced using data files with only epochs marked as non-wearing removed. The current Table 4 was produced using data files with both non-wearing and discarded epochs removed so that visual identification and DT could be compared on the same epochs (minutes). These discarded epochs were predominantly marked as bedrest by the DT. Therefore, the removal of these epochs from analysis reduced the average bedrest (min/day) to that seen in the new Table 4.  As recommended by the Reviewer, the corresponding parts in the manuscript text were modified.

Lines 144-145: *The software enabled the raters to discard data at the beginning and end of each Actigraph file.*

Lines 290-292: *Bedrest characteristics were performed after epochs (minutes) of not-wearing and discarded in visual identification were removed.*

Lines 352-356: *As identified by DT and shown in Table 4, the average daily bedrest time was over 9 hours (502 ± 62 min/day), ranging from almost 5 hours (294 min/day) to over 10 hours (652 min/day). Mean sleep duration obtained from the parental survey was significantly longer than bedrest identified by DT (650 and 502 min/day; P<0.001), Sadeh’s algorithm (650 and 604 min/day; P<0.001),* *and visual identification (650 and 502 min/day; P<0.001).*

Lines 371-376: *On average, the wake time identified by DT was over 11 hours (734 min/day) and was longer than Sadeh's algorithm (693 min/day). The number of children taking naps reported in the parental survey (n=45) was lower than the DT identified (n=134). Naps were identified by DT in 67% of the children at least once during the monitoring period. In children whose parents reported taking daytime naps daily (23%), the naps frequency was higher than those identified by DT (1.05 ± 0.27 and 0.25 ± 0.24).*
